# Rapid Flow Cytometry-Based Test for the Diagnosis of Lipopolysaccharide Responsive Beige-Like Anchor (LRBA) Deficiency

**DOI:** 10.3389/fimmu.2018.00720

**Published:** 2018-04-23

**Authors:** Laura Gámez-Díaz, Elena C. Sigmund, Veronika Reiser, Werner Vach, Sophie Jung, Bodo Grimbacher

**Affiliations:** ^1^Center for Chronic Immunodeficiency, University Medical Center Freiburg, Freiburg, Germany; ^2^Faculty of Biology, University of Freiburg, Freiburg, Germany; ^3^Institute of Medical Biometry and Statistics, Faculty of Medicine, University Medical Center Freiburg, Freiburg, Germany; ^4^Pôle de Médecine et de Chirurgie Bucco-Dentaires, University Hospital, Faculty of Dentistry, University of Strasbourg, Strasbourg, France; ^5^Institute of Immunology and Transplantation, Royal Free Hospital, University College London, London, United Kingdom

**Keywords:** LRBA, flow cytometry, diagnosis, test, PID, ROC

## Abstract

The diagnosis of lipopolysaccharide-responsive beige-like-anchor-protein (LRBA) deficiency currently relies on gene sequencing approaches that do not support a timely diagnosis and clinical management. We developed a rapid and sensitive test for clinical implementation based on the detection of LRBA protein by flow cytometry in peripheral blood cells after stimulation. LRBA protein was assessed in a prospective cohort of 54 healthy donors and 57 patients suspected of LRBA deficiency. Receiver operating characteristics analysis suggested an LRBA:MFI ratio cutoff point of 2.6 to identify LRBA-deficient patients by FACS with 94% sensitivity and 80% specificity and to discriminate them from patients with a similar clinical picture but other disease-causing mutations. This easy flow cytometry-based assay allows a fast screening of patients with suspicion of LRBA deficiency reducing therefore the number of patients requiring LRBA sequencing and accelerating the treatment implementation. Detection of biallelic mutations in *LRBA* is however required for a definitive diagnosis.

## Introduction

Primary immunodeficiencies (PIDs) are genetic disorders of the immune system with heterogeneous and variable clinical phenotypes. Advances in genetics and molecular biology have allowed researchers to identify more than 300 genes associated with different PIDs phenotypes (1). The lipopolysaccharide-responsive beige-like anchor protein (LRBA) deficiency is a PID caused by the loss of LRBA protein expression, due to either homozygous or compound heterozygous mutations in *LRBA* (2). Similar to eight other human proteins such as lysosomal-trafficking regulator (LYST) and neurobeachin, LRBA contains a BEige And Chediak-Higashi (BEACH) domain located between a PH-like domain important for membrane association, and a WD-40 domain responsible for ligand binding and signaling (3). BEACH domain-containing proteins (BDCPs) are highly conserved among species and are widely expressed in human tissues (3). In 2001 and 2004, LRBA was reported to be expressed in murine monocytes after stimulation with lipopolysaccharide (LPS) and to be overexpressed in breast and prostate cancer tissues (4, 5). Although the worldwide prevalence of LRBA deficiency is still unknown, we and others have diagnosed a total of more than 80 patients (2, 6–17). These patients show a wide spectrum of clinical manifestations including autoimmune manifestations, enteropathy, and organomegaly, as well as with hypogammaglobulinemia leading to recurrent pulmonary infections (2, 6–14). The clinical management typically includes immunosuppressants, immunoglobulin replacement, antibiotics, and some patients required hematopoietic stem cell transplantation (HSCT) (14, 15). Generally, patients diagnosed at early stages of the disease have a better prognosis and an improved quality of life due to an earlier and more accurate management. This calls for a prompt diagnosis of patients. However, the diagnosis of LRBA deficiency currently relies on gene sequencing approaches that are expensive, time-consuming, and that are not considered as routine technologies in most laboratories (2, 6–15). Therefore, the development of an accurate, rapid, sensitive, and cost-effective test that allows diagnosing LRBA deficiency is warranted. Here, we describe a flow cytometry-based assay as a reliable tool for the screening of LRBA deficiency based on the detection of LRBA expression in stimulated peripheral blood mononuclear cells (PBMCs).

## Materials and Methods

### Samples

Peripheral blood mononuclear cells were extracted from EDTA or heparinized blood of adult healthy volunteers, and from patients suspected of LRBA deficiency that were referred to the Center of Chronic Immunodeficiency (CCI, Freiburg, Germany), by density gradient centrifugation over Lymphoprep^TM^ density gradient medium (Axis-Shield; cat. No. 1114545). For inclusion criteria, please refer to Gámez-Díaz et al. (17), or to Table S1 in Supplementary Material. Epstein–Barr virus transformed B cells (EBV cells) were generated from MACS beads-isolated peripheral blood B cells as described before by Hui-Yuen et al. (18). The scientific committee at the University Medical Center of Freiburg approved this research on the basis of a written informed consent (Approval No. 290/13).

### Stimulation Conditions

Fresh or frozen PBMC, and sorted PBMC subsets were cultured as 250000 cells in 96-well plates in complete RPMI medium (RPMI-1640; Gibco) supplemented with 100 U/ml penicillin, 100 µg/ml streptomycin (Sigma-Aldrich; cat. no. 15140122), and 10% fetal calf serum (Gibco; 10082139). Cells were stimulated with 20 ng/ml PMA (phorbol 12-myristate 13-acetate) (Sigma-Aldrich; cat. no. P1585), 1 µg/ml ionomycin (Sigma-Aldrich; cat. no. I0634), Dynabeads^TM^ Human T-Activator CD3/CD28 (Life Technologies cat. no. 1131D), 10 ng/ml phytohemagglutinin (PHA) (Sigma-Aldrich; cat. no. L1668), 1 µg/ml staphylococcal enterotoxin B (SEB; Sigma-Aldrich; cat. no. S4881), 10 µg/ml pokeweed mitogen (PWM; Sigma-Aldrich; cat. no. L8777), *Staphylococcus aureus* Cowan I (SAC mitogen; Sigma-Aldrich; cat. No. 82526), CD40 + IL-21 (kind gift of Prof. Hermann Eibel, CCI; Freiburg), 100 ng/ml anti-human IgM (Southern Biotech; cat. No. 2020-01), 100 U/ml recombinant human IL-2 (ImmunoTools; cat. no. 11340025), 100 µM CpG (Invivogen, cat. no. tlrl-2006), 10 ng/ml IFN-alpha (Preprotech, cat. no.306-02A), or 10 ng/ml LPS (Sigma-Aldrich; cat. no. L4391).

### Detection of LRBA by Flow Cytometry

Before addition of the stimuli (day 0) and following stimulation, intracellular expression of LRBA was determined as follows: 250000 cells were first resuspended in 50 µl of PBS and stained for extracellular markers for 15 min at 4°C with anti-CD19-PE-Cy7 (BD; cat. no. 341113, dilution 1:100) CD3-APC-H7 (BD; cat. no. 555342, dilution 1:300), CD69-FITC (BD; cat. no. 555530, 1:20), CD16-PE-Cy5 (BD; cat. no. 555408, dilution 1:20), CD56-PE-Cy5 (BD; cat. no. 555517, dilution 1:300), CD14-APC (BD; cat. no. 555399, dilution 1:10), and with fixable viability dye (FVD eFluor506, eBioscience; cat. no. 65-0866, dilution 1:500). Next, the cells were fixed and permeabilized for 20 min using 200 µl of BD Cytofix/Cytoperm solution^TM^ (BD; cat. no. 554715), and then washed twice at 1700 rpm during 2 min with 200 µl of 1× Perm/Wash Buffer^TM^ (BD; cat. no. 554723). Subsequently, cells were resuspended in 50 µl of 1× Perm/Wash Buffer and stained with a rabbit polyclonal anti-LRBA antibody (Sigma; cat. no. HPA023597 or HPA019366, dilution 1:400) for 30 min at 4°C. A secondary antibody F(ab′)2 donkey anti-rabbit IgG-PE (BD; cat. no. 558416, dilution 1:25) was added and incubated at 4°C for 30 min. Cells were then washed and acquired on a FACS Canto II (BD). Data analysis and calculation of the geometric mean fluorescence intensity (MFI) were performed using FlowJo^TM^ 7.6.5 software (TreeStar Inc., Ashland, OR, USA). Gating strategy was done as follows: cells were first gated for mononuclear cells (SSC-A vs. FSC-A) and singlets (FSC-H vs. FSC-A). Then, living mononuclear cells were gated based on the negative staining for the FVD and separated in cell subsets according to their extracellular markers. Cell activation was confirmed by measuring CD69 expression. Finally, LRBA expression was analyzed as univariate histogram. EBV cells were also stained and analyzed as described for PBMCs.

### Western Blotting

Protein lysates were size-fractionated by SDS-PAGE (12, 8, and 4% gradient gel), electrotransferred to a PVDF membrane for 1.5 h at 45 V and immunodetected with a rabbit polyclonal anti-LRBA antibody (Sigma-Aldrich; cat. no. HPA023597). The specific binding of the antibody to the LRBA protein was detected with a secondary HRP anti-rabbit IgG (Cell signaling; cat. no. 7074) as a band of ~319 kDa. Tubulin (Abcam; cat. no. 4074) was used as protein loading control, and was detected as a band of ~50 kDa.

### Next-Generation Sequencing (NGS)

Briefly, 225 ng of gDNA was digested and hybridized with a HaloPlex biotinylated probe library in presence of an indexing primer cassette for enrichment. After capturing and ligating the circularized target DNA-probe hybrids with streptavidin beads, amplification of targeted fragments was done by PCR. Sample barcodes were introduced during amplification for precise tracking. After elution, PCR-amplification and pooling of equimolar amounts of indexed targeted-samples were prepared for multiplexed sequencing on the Illumina MiSeq platform. Having run samples of different quantity and quality, we averaged 99% of the bases in the exons covered with a depth of at least 38-fold. The sequences were aligned to the human genome using the Agilent SureCall^TM^ software.

### Sanger Sequencing

All variants detected by NGS were validated using Sanger sequencing. LRBA exons were amplified by PCR from gDNA according to standard protocols. Primer sequences and PCR conditions are available upon request. The PCR products were sequenced in both directions by Sanger sequencing and analyzed with Sequencher version 4.10.1. (Genes Codes Corporation).

### Statistical Analysis

We performed a cross-sectional study with prospective data collection to illustrate the operative characteristics by receiver operating characteristic (ROC) analysis of our flow cytometry-based test for the diagnosis of LRBA deficiency. *LRBA* sequencing results were used as gold standard. Optimal MFI ratio cut-point of LRBA to distinguish between “mutated (definitive) *LRBA* patients” and “wild-type (non) *LRBA* patients” was selected among all potential cut-points values, considering a minimum of 90% sensitivity and maximal specificity. ROC statistical analysis was performed using StataCorp. 2015 (Stata Statistical Software: Release 14. College station, TX: StataCorp LP). Statistical significances were calculated with a non-parametric two-tailed Mann–Whitney test using GraphPad Prism 6.0 software. A *p* value of <0.05 was considered statistically significant (*p < 0.05; **p < 0.01; ***p < 0.001; ****p < 0.0001).

### Calculation of the LRBA-MFI Ratio

The LRBA-MFI ratio is calculated separately for healthy donors and patients as follows:

$$\text{LRBA-MFI ratio}=\frac{\begin{array}{l} \text{MFI of LRBA expression on PHA}\\ \;-\text{stimulated PBMCs} \end{array}}{\begin{array}{l} \text{MFI of LRBA expression on }\\ \text{ unstimulated PBMCs} \end{array}}$$

## Results

In order to confirm the sensitivity of this test and the accuracy of two different LRBA antibodies commercially available, LRBA expression was measured in EBV cell lines from a healthy donor and a patient (P1) bearing a homozygous splice site mutation (c.2004 + 2A > G) in *LRBA* (14, 17). Healthy donor EBV cells constitutively express LRBA protein as shown in Figure S1 in Supplementary Material and in Lopez-Herrera et al. (2), while P1 failed to express LRBA protein. Both antibodies showed specific binding to intracellular LRBA (Figure S1A in Supplementary Material) that was lost when adding a specific LRBA-blocking peptide in a dose-dependent manner (Figure S1B in Supplementary Material).

**Figure 1:**
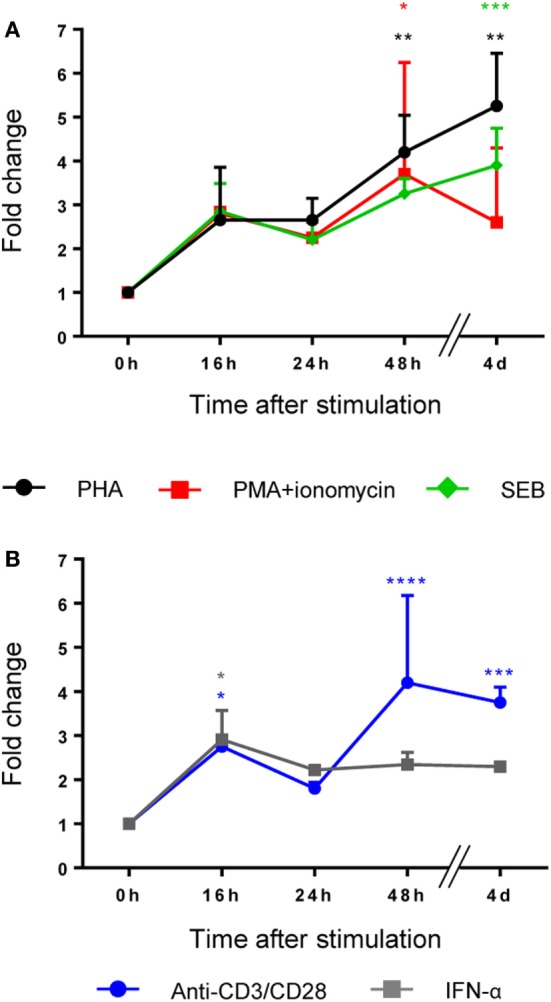
A broad panel of stimuli promotes the expression of LRBA protein in total peripheral blood mononuclear cells (PBMCs). PBMCs from healthy donors were stimulated with **(A)** phytohaemagglutinin (PHA), PMA + ionomycin and staphylococcal enterotoxin B (SEB) **(B)** anti-CD3/anti-CD28 and IFN-α, during 16, 24, 48 h, and 4 days. Values are presented as mean fluorescence intensity fold changes from time 0 h which was normalized to one. All stimulation conditions and time points were performed in three samples independently. Statistical analysis was done comparing 0 h with 16, 24, 48, or 4 days, respectively, after stimulation.

**Figure 2:**
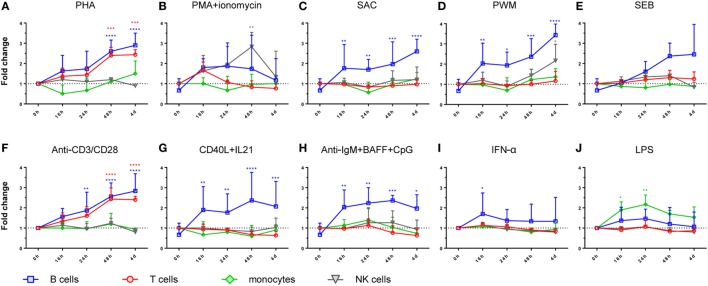
Time course of LRBA protein expression in peripheral blood mononuclear cells subsets. B-lymphocytes, T-lymphocytes, NK cells, and monocytes are represented in blue, orange, green, and gray, respectively. Values are presented as mean fluorescence intensity fold changes from time 0 h, which was normalized to one. Statistical analysis was done comparing 0 h with 16, 24, 48, or 4 days, respectively, after stimulation with **(A)** phytohaemagglutinin (PHA), **(B)** PMA + ionomycin, **(C)** SAC, **(D)** pokeweed mitogen (PWM), **(E)** staphylococcal enterotoxin B (SEB), **(F)** anti-CD3/anti-CD28, **(G)** CD40L + IL-21, **(H)** anti-IgM + BAFF + CpG, **(I)** IFN-alpha, and **(J)** lipopolysaccharide (LPS). Statistical significance of the different cell subsets are represented with the same color as the cell subpopulation, i.e., blue, orange, green, and gray, for B-lymphocytes, T-lymphocytes, monocytes, and NK cells, respectively. All stimulation conditions and time points were performed in three samples independently.

LRBA protein is widely expressed in human tissues (2, 5). However, PBMCs need to be stimulated to promote the expression of LRBA (2, 5). Therefore, we performed a time course assay with different cell-type specific stimuli (mitogens or receptor-specific stimuli) in order to determine the best time point and the best stimulation condition that promotes LRBA expression in total human PBMCs (Figure 1) and in specific PBMC subsets (Figure 2). Flow cytometry analysis revealed that total PBMCs showed the highest expression of LRBA upon stimulation with PHA for four days. However, stimulation with anti-CD3/anti-CD28 or with PMA along with ionomycin for 2 days, or SEB for 4 days, also led to high LRBA protein synthesis (Figure 1A). In addition, stimulation with anti-CD3/anti-CD28 or IFN-α produced the earliest significant LRBA expression, observed after 16 h (Figure 1B). Stimulation with PWM, SAC, LPS, and cocktails with IgM + BAFF + CpG, or CD40L + IL-21 did not induce significant LRBA expression in total PBMCs (Figures S2A,B in Supplementary Material).

Moreover, all PBMC subsets expressed LRBA after stimulation. Particularly, B lymphocytes showed the highest expression after 4 days of stimulation with PWM, PHA, or anti-CD3/anti-CD28, followed by 3 days of stimulation with CD40 + IL-21, or IgM + CpG + BAFF (Figures 2A,F,G,H). T lymphocytes exhibited higher LRBA expression with PHA, or with anti-CD3/anti-CD28 (Figures 2A,F). In monocytes and NK cells, the peak of LRBA expression occurred after 24 h of stimulation with LPS or a 2-day stimulation with PMA + ionomycin, respectively (Figures 2J,B).

Following the results of our time course assay, the lowest variability and the strongest LRBA expression was observed in PHA-stimulated cells. This observation was confirmed by testing PBMCs from seven healthy donors (Figures S3A,B in Supplementary Material). Additionally, LRBA expression was not affected by the type of anticoagulant used during blood withdrawal (EDTA or heparin), by the time between blood withdrawal and analysis (fresh, 12, 24, 48, and 72 h), or by storage conditions (fresh and frozen cells) (Figures S4A,B in Supplementary Material, respectively).

**Figure 3:**
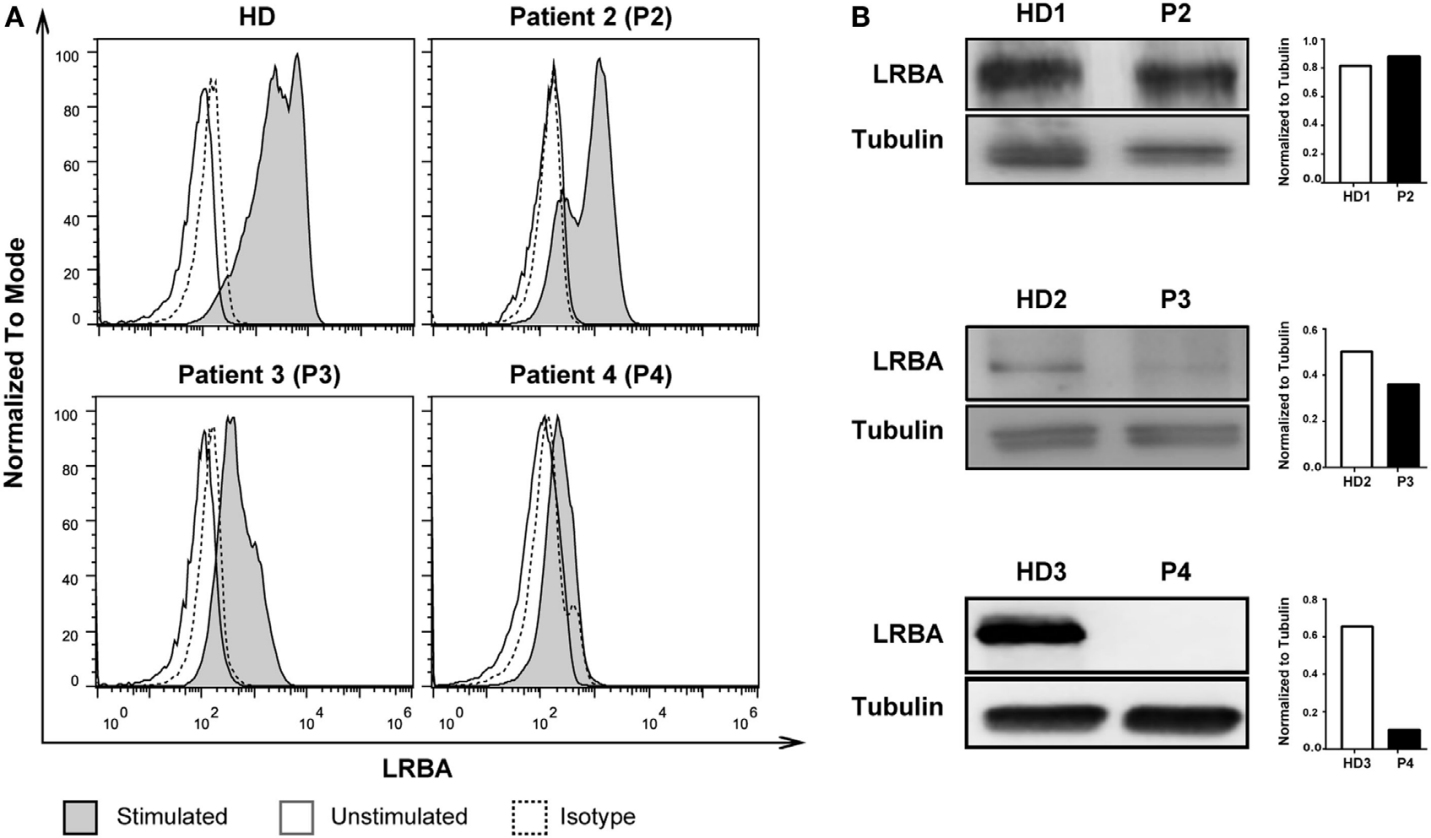
Comparison of LRBA protein expression detection using flow cytometry and western blotting. **(A)** Flow cytometry analysis of phytohaemagglutinin (PHA)-stimulated peripheral blood mononuclear cells showing normal LRBA expression in a healthy donor (HD) and in patient 2 (P2); reduced LRBA expression in patient 3 (P3); and absence of LRBA expression in patient 4 (P4). **(B)** Detection of LRBA protein (~319 kDa) and tubulin (~50 kDa) in PHA-stimulated cell lysates from HD, P1, P2, P3, and P4. Respective densitometry quantification graphs of LRBA protein expression normalized against tubulin is shown next to each blot.

We next compared the accuracy of the flow cytometry vs. the Western Blot approach for LRBA expression detection by testing stimulated PBMCs from a healthy control and three patients with suspected of LRBA deficiency (P2, P3, and P4). The LRBA expression level determined by flow cytometry was assessed by calculating the ratio of LRBA geometric mean fluorescence intensity (MFI) of stimulated vs. unstimulated total PBMCs. Our results showed that patient P2 had normal LRBA expression (MFI ratio: 2.6), patient P3 had a severely reduced expression (MFI ratio: 1.4), and patient P4 had an absence of LRBA protein expression (MFI ratio: 1.1) as demonstrated both by flow cytometry and western blotting (Figures 3A,B; Table 1). Consecutive *LRBA* sequencing demonstrated wild-type *LRBA* sequence for patient P2 but a homozygous (p.T2388Pfs*7), and a compound heterozygous mutation in *LRBA* (p.Q473*/p.E945Efs*) in patients P3 and P4, respectively (17).

**Table 1:** Mean fluorescence intensity (MFI) ratios of LRBA protein expression between patients with different LRBA diagnosis.

Sample	MFI ratio^a^	Diagnosis
Healthy donor	3.3	Normal
Patient 2	2.6	Normal
Patient 3	1.4	Reduced
Patient 4	1.1	Absence

*^a^MFI ratio was calculated as LRBA-MFI in stimulated cells divided by LRBA-MFI in unstimulated cells*.

**Figure 4:**
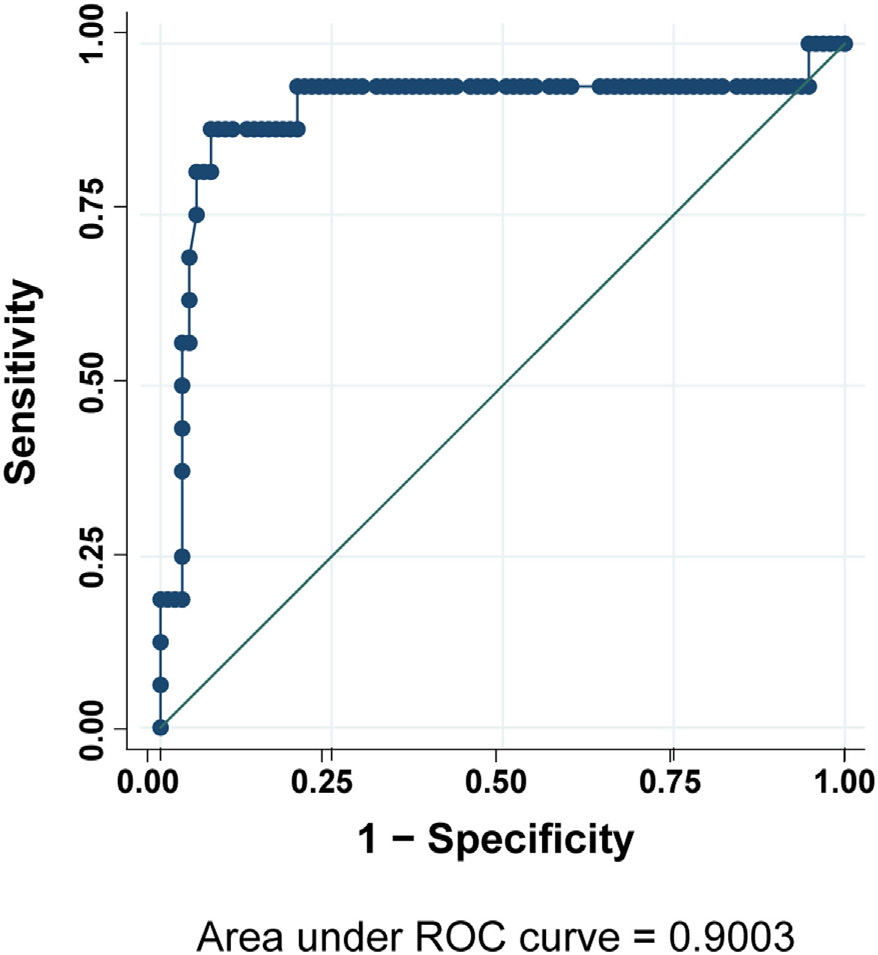
Receiver operating characteristic (ROC) curve analysis for LRBA protein expression. Analysis was done on 57 sequenced patients and 54 healthy donors.

**Figure 5:**
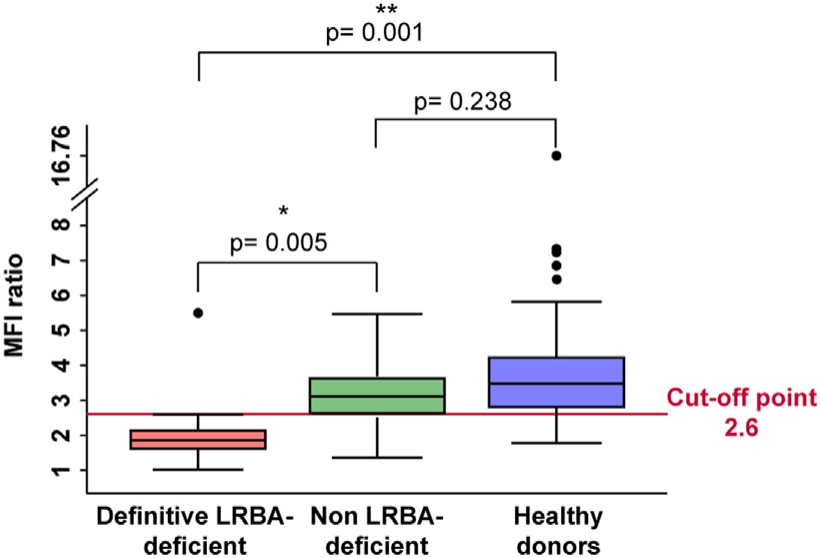
LRBA deficient patients have significantly reduced mean fluorescence intensity (MFI) ratio of LRBA expression in peripheral blood mononuclear cells compared to healthy donors. Boxplot analysis showed that definitive LRBA deficient- patients represented in red, have significantly reduced MFI ratio of LRBA expression compared to healthy donors represented in blue (*p* = 0.001) and to non-LRBA deficient patients represented in green (*p* = 0.005). Each value bigger or smaller than this range is marked as a black dot.

According to these results, we analyzed by flow cytometry and further *LRBA* sequencing all patients with a clinical suspicion of LRBA deficiency (or known as possible LRBA-deficient patients) that were referred to our center. To date, we have evaluated a total of 111 individuals corresponding to 54 healthy donors (15 were healthy family members) and 57 patients. Amongst them, 16 patients had biallelic mutations in *LRBA* revealed by targeted re-sequencing of 77 common variable immunodeficiency (CVID)-associated genes. These patients were classified as “definitive LRBA-deficient.” The mutations identified in *LRBA* include eight different homozygous mutations present in 10 patients, and 9 compound heterozygous mutations in 6 additional patients (Table S2 in Supplementary Material). Eight of these 16 patients have already been published by our group (17). The remaining 41 patients had wild-type *LRBA* sequence and therefore were classified as non-LRBA-deficient. In order to discriminate LRBA-deficient patients from non-LRBA deficient patients and from healthy donors using our flow cytometry-based test, we performed a ROC analysis comparing all three groups of individuals from our cohort (16 patients with mutations in *LRBA*, 41 wild-type *LRBA* patients, and 54 healthy donors). The ROC curve generated an area under the curve of 0.90 with a 95% CI of (0.75, 1.00) (Figure 4). To reach a sensitivity of 94%, we selected a LRBA-MFI ratio cut-point of 2.6, which was associated with a specificity of 80%. According to our cohort, 81.5% of healthy donors (44 individuals) showed an LRBA-MFI ratio higher than 2.6 with a mean of 4.1. Thirty-two wild-type *LRBA* patients (78%) presented a LRBA-MFI ratio mean of 3.5, whereas nine of them presented an LRBA-MFI ratio below 2.6. Conversely, 15 out of the 16 patients with biallelic mutations in *LRBA* were characterized by a ratio below 2.6 (mean 1.7) (Table 2; Figure S5 in Supplementary Material). Individuals with suboptimal cell activation after stimulation, which was defined as ≤10% of CD69 positive cells, were excluded from the analyses in order to avoid false negatives, since LRBA expression directly depends on cell activation. Thus, with an LRBA-MFI ratio of 2.6, we were able to discriminate between healthy donors and definitive LRBA-deficient patients (*p* = 0.001), and between non LRBA-deficient patients and definitive LRBA-deficient patients (*p* = 0.005), see Figure 5.

**Table 2:** Mean fluorescence intensity (MFI) in healthy donors and patients suspected of LRBA deficiency.

Groups	Number of patients	MFI ratio^a^ (STD)	Range (min–max)
Healthy donors	54	3.85 (1.56)	2.14–10
Definitive LRBA-deficient patients	16	1.96 (1.05)	0.83–5.5
Not LRBA-deficient patients	41	3.18 (0.90)	1.25–5.23

*^a^MFI ratio was calculated as MFI of stimulated cells divided by MFI of unstimulated cells. Classification was done based on LRBA sequencing results*.

## Discussion

Here, we demonstrate that a flow cytometry-based assay is a valuable tool for investigating LRBA deficiency. Currently, the screening method for LRBA deficiency is based on gene sequencing approaches, or in some cases, on protein detection by Western Blot (1–8). On one hand, genome-wide approaches are expensive, time-consuming, and are not performed in conventional diagnostic laboratories. Additionally, the *LRBA* gene contains 58 exons, making Sanger sequencing a non-ideal option. On the other hand, LRBA-Western Blotting is a complex technique since LRBA is a high-molecular-weight protein of 319 kDa. The major challenge is the difficulty to detect LRBA by Western Blotting even in stimulated samples from healthy donors. This may lead to misinterpretation of the results and several repetitions of the test are often required. Our LRBA flow cytometry-based assay is however an easier and faster approach that also enables at the same time to perform a cellular immunophenotyping, as LRBA intracellular staining can be combined with staining of other extracellular or intracellular antigens.

Among a large panel of stimuli, including antigen stimulation, mitogens, bacterial superantigens, cytokines, and toll-like receptors agonists, the most efficient activation condition promoting the expression of LRBA in total PBMCs was the stimulation with PHA for 4 days followed by the stimulation with anti-CD3/anti-CD28 for 48 h. Despite the fact that all PBMC subsets expressed LRBA protein under different stimulation conditions, B-lymphocytes showed the highest expression after PWM and PHA stimulation (9). This calls for future research aimed at identifying the specific role of LRBA in B-lymphocytes to allow a better understanding of the pathomechanisms of LRBA deficiency. However, stimulation of sorted cell subsets as well as an evaluation of mRNA expression would be necessary to confirm our findings. Nevertheless, we demonstrated here the availability of a broad range of stimuli that triggers LRBA expression in PBMCs, increasing the implementation of LRBA-testing in diagnostics centers worldwide. In addition, we observed that LRBA expression was not affected by the type of anticoagulants used (EDTA or heparin), or after 48 h of cell fixation, as reported for other activation markers (19). LRBA expression was also comparable in freshly isolated PBMCs; 24, 48, and 72 h after blood withdrawal or after thawing of cryopreserved PBMCs. Cryopreservation facilitates the shipment of PBMCs to diagnostic laboratories and allows the storage in liquid nitrogen for the evaluation of several samples at the same time.

The LRBA expression level was assessed by calculating LRBA geometric MFI ratio in stimulated vs. unstimulated total PBMCs. According to our results, most healthy individuals showed an LRBA-MFI ratio higher than 2.6, whereas patients that have been diagnosed as “definitive” LRBA deficiency were characterized by a stimulation ratio below 2.6. However, one patient from our cohort who bears a homozygous mutation in *LRBA* (W225*) has a normal LRBA protein expression. Since this mutation has been described for the first time here, we cannot exclude that there is a residual protein expression in the patient despite the homozygous stop codon, e.g., by readthrough mechanisms. Indeed, LRBA-deficient patients with residual LRBA expression have been also reported by others (10, 16). We found 10 healthy donors, including 2 family members with LRBA MFI ratios below 2.6, despite optimal cell activation (measured by CD69). Nevertheless, their LRBA expression was higher than their paired LRBA patient sample when the difference of the LRBA MFI ratios (LRBA MFI ratio from healthy donor – LRBA MFI ratio from patient) was calculated (data not shown). Finally, nine patients with decreased expression of LRBA have a wild-type *LRBA* sequence. This reduced expression might be a consequence of mutations in other genes belonging to the functional pathway of LRBA. Further analysis of the CTLA-4 shuttling and trans-endocytosis assays should be considered as functional tests in patients with possible residual LRBA expression, as it has been recently shown that LRBA affects CTLA-4 trafficking and lysosomal degradation through direct protein–protein interaction (9, 20).

Taken together, an LRBA-MFI ratio higher than 2.6 generally suggests the absence of an LRBA-related mutation. However, due to the intrinsic limitations of flow cytometric studies, this approach does not exclude protein positive LRBA deficiency and further studies are still warranted in patients where LRBA deficiency suspicion is high. The assessment of LRBA expression should be performed by calculating geometric MFI ratios, since the fluorescence intensity can vary between experiments, particularly after repeated thawing and freezing cycles of the anti-LRBA antibody (data not shown). In addition, the LRBA-MFI ratio of stimulated vs. unstimulated PBMCs of any given individual must always be compared to a MFI ratio of a matched healthy/travel control. In addition, a difference of LRBA MFI ratio of the healthy donor and its paired patient sample should be calculated when both individuals present LRBA MFI ratios below 2.6. Moreover, the expression of a cell activation marker, such as CD69, for example, should always be included in the staining controls in order to confirm the efficiency of the cell stimulation step. The reported sensitivity and specificity of this flow cytometry test has to be taken with care as they are based on a selected sample collection.

The LRBA flow cytometry assay has been used for 3 years as a routine test at the Center for Chronic Immunodeficiency in Freiburg, Germany, for screening patients with a clinical suspicion of LRBA deficiency, including cohorts with autoimmune lymphoproliferative syndrome (ALPS), CVID, child-onset hypogammaglobulinemia, and immunodysregulation polyendocrinopathy enteropathy X-linked-like syndrome (IPEX-like). Since residual LRBA protein expression can be detected in some LRBA-deficient patients, physicians might consider *LRBA* sequencing not only in patients with an absence of LRBA protein, but also in patients with severe reduced LRBA protein expression, or with a MFI ratio below 2.6 (12, 14). We therefore suggest that the detection of biallelic mutations in *LRBA* by sequencing is required to provide a “definitive” diagnosis of LRBA deficiency. However, our FACS test allows narrowing the cohort of patients that need further *LRBA* sequencing, which according to the experience of our diagnostic center in Freiburg, costs 250 EUR per sample, whereas the costs of LRBA Sanger sequencing or targeted-panel sequencing are 3,500 EUR (50 EUR per exon) and 2,900 EUR, respectively. These observations were the basis of the present study, where we see the need to extent our LRBA-flow cytometry test to the other diagnostic centers that cannot afford LRBA sequencing. In addition, a flow cytometry test can be performed in conventional (mid-level) diagnostic laboratories, whereas gene sequencing obviously needs sequencing technology which is not readily available at immunology centers worldwide. Finally, in addition to the diagnostic value of our LRBA-flow cytometry analysis, our test can also be a useful tool for evaluating and monitoring LRBA expression after HSCT. The efficacy of HSCT is not clear in the context of this disease, since only few LRBA-deficient patients have received this treatment (11, 14, 15).

Therefore, according to our clinical and laboratory experience on LRBA deficiency, physicians should suspect LRBA deficiency when the following criteria are met: (i) disease onset before the age of 12 years and (ii) presence of autoimmunity, lymphoproliferation, severe colitis alone or in association with hypogammaglobulinemia and recurrent infections. If the patients meet the above criteria, LRBA protein expression should be assessed by flow cytometry in PBMCs after stimulation. If the MFI ratio is lower than 2.6 and the MFI difference according to the paired healthy donor is higher than 1.0 after proven cell activation, then *LRBA* should be sequenced to confirm the diagnosis. If the sequencing reveals wild-type *LRBA*, then CTLA-4 expression, CD80–CD86 trans-endocytosis assays or *CTLA-4* sequencing should be next addressed. The latter is recommended due to the high clinical similarity between LRBA deficiency and CTLA-4 insufficiency (21).

In conclusion, we provide a reliable protocol to screen for LRBA deficiency using flow cytometry, a routine easy-to-use technology that is currently implemented for many other immunological tests. LRBA protein detection by flow cytometry will help to reduce the diagnostic delay, allowing timely clinical interventions.

## Ethics Statement

Ethik-Kommission der Albert-Ludwigs-Universität Freiburg. The scientific committee at the University Medical Center Freiburg approved this research on the basis of a written informed consent (Approval No. 290/13).

## Author Contributions

LG-D and BG designed the study. LG-D and ES performed the experiments. LG-D, VR, WV, and SJ analyzed the data. LG-D and BG wrote the manuscript. LG-D, VR, WV, and SJ prepared the figures. All authors reviewed and approved the manuscript.

## Conflict of Interest Statement

The authors declare that the research was conducted in the absence of any commercial or financial relationships that could be construed as a potential conflict of interest.

## Supplementary Material

Figure S1 Detection of LRBA protein by flow cytometry **(A)** LRBA protein was detected in Epstein–Barr virus (EBV) cells from healthy donor (blue), LRBA-deficient patient (red), isotype control (dotted line), using anti-LRBA antibody SIGMA HPA019366 (right) and anti-LRBA antibody SIGMA HPA023597 (left). **(B)** EBV cells from healthy donor (blue) treated with LRBA-blocking peptide (Atlas Antibodies, cat. no. APrEST74668) using different ratios of peptide: LRBA antibody.

Figure S2 Time course of LRBA protein expression in total peripheral blood mononuclear cells (PBMCs) using different stimuli. Statistical analysis was performed comparing 0 h with 16, 24, 48 h, or 4 days after stimulation. All stimulation conditions and time points were performed in three samples independently.

Figure S3 Peripheral blood mononuclear cells (PBMCs) need stimulation to promote LRBA expression. LRBA expression was detected by flow cytometry in fresh PBMCs from seven different healthy donors without stimulation (white squares) and upon stimulation with phytohaemagglutinin (PHA) (10 ng/µl) for 3 days (black squares) **(A)**, or by western blotting in fresh PBMCs from three healthy donors before and after stimulation with PHA **(B)**.

Figure S4 LRBA expression is not affected by **(A)** the type of anticoagulant use for blood withdrawal, by the time of withdrawal, or **(B)** by frozen storage.

Figure S5 Scatter plots representing the LRBA-MFI ratio distribution of the different 16 definitive LRBA patients (red dots), and 41 non-LRBA patients (green dots) according to their respective healthy donors (blue dots).

Table S1 Classification of patients with a suspicion of LRBA deficiency.

Table S2 *LRBA* mutations identified in our patient’s cohort.

## Abbreviations

ALPS, autoimmune lymphoproliferative syndrome; CVID, common variable immunodeficiency; EBV, Epstein–Barr virus; HSCT, hematopoietic stem cell transplantation; IBD, inflammatory bowel disease; IPEX, immunodysregulation polyendocrinopathy enteropathy X-linked; MFI, mean fluorescence intensity; LRBA, lipopolysaccharide-responsive beige-like anchor protein; LPS, lipopolysaccharide; NK, natural killer; PBMC, peripheral blood mononuclear cells; PHA, phytohaemagglutinin; PID, primary immunodeficiency; PMA, phorbol myristate acetate; PWM, pokeweed mitogen; ROC, receiver operating characteristic; SAC, *Staphylococcus aureus* Cowan I; SEB, staphylococcal enterotoxin B; LYST, lysosomal-trafficking regulator; BEACH, BEige And Chediak-Higashi; BDCP, BEACH domain-containing proteins.
