# Supplementary figures and images for: Rapid Flow Cytometry-Based Test for the Diagnosis of Lipopolysaccharide Responsive Beige-Like Anchor (LRBA) Deficiency
Source: Front Immunol. 2018 Apr 23;9:720. doi: 10.3389/fimmu.2018.00720 (PMC5925005; doi:10.3389/fimmu.2018.00720)

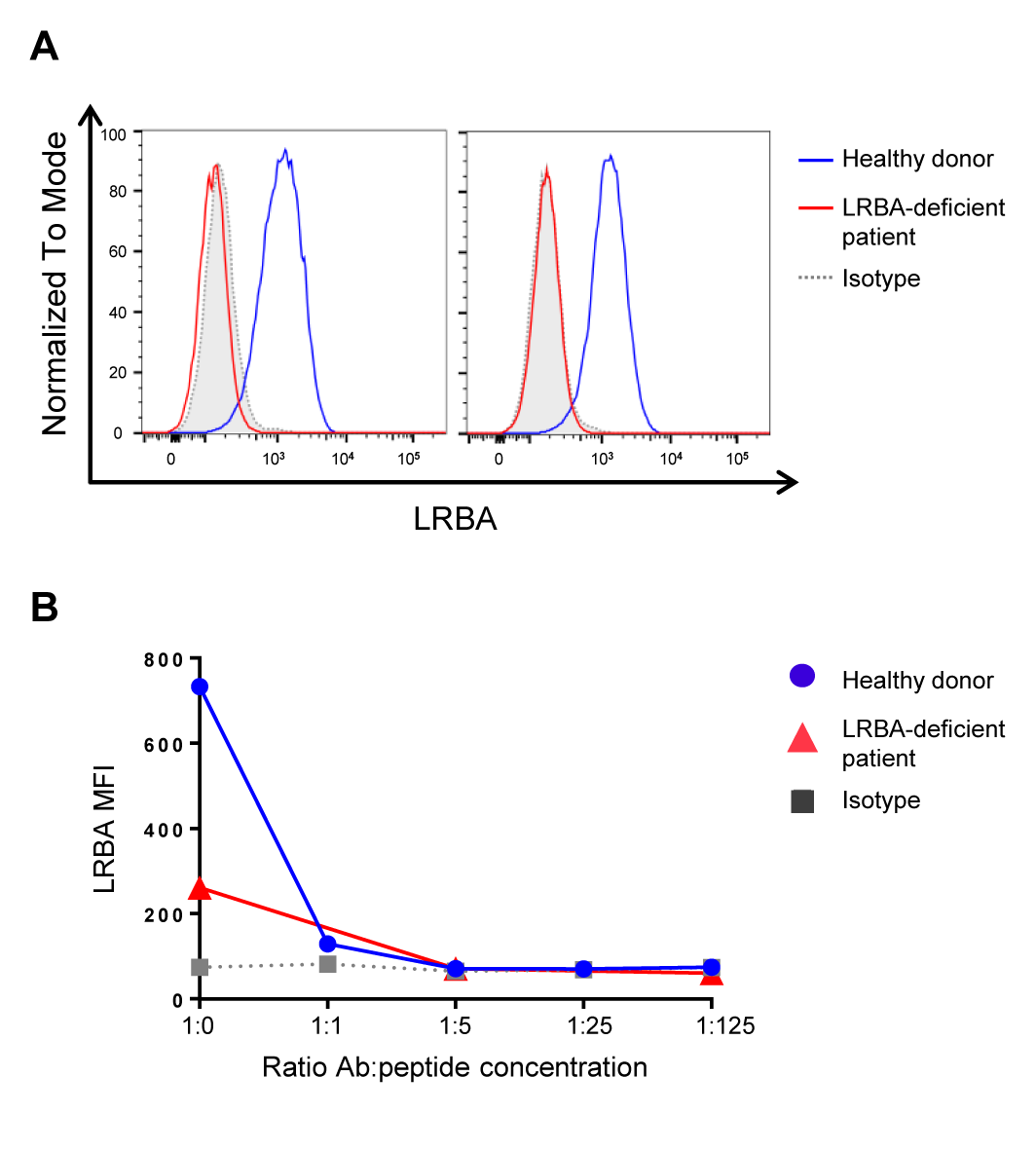

Supplement: Figure S1 — Detection of LRBA protein by flow cytometry (A) LRBA protein was detected in Epstein–Barr virus (EBV) cells from healthy donor (blue), LRBA-deficient patient (red), isotype control (dotted line), using anti-LRBA antibody SIGMA HPA019366 (right) and anti-LRBA antibody SIGMA HPA023597 (left). (B) EBV cells from healthy donor (blue) treated with LRBA-blocking peptide (Atlas Antibodies, cat. no. APrEST74668) using different ratios of peptide: LRBA antibody. [file image_1.tif]

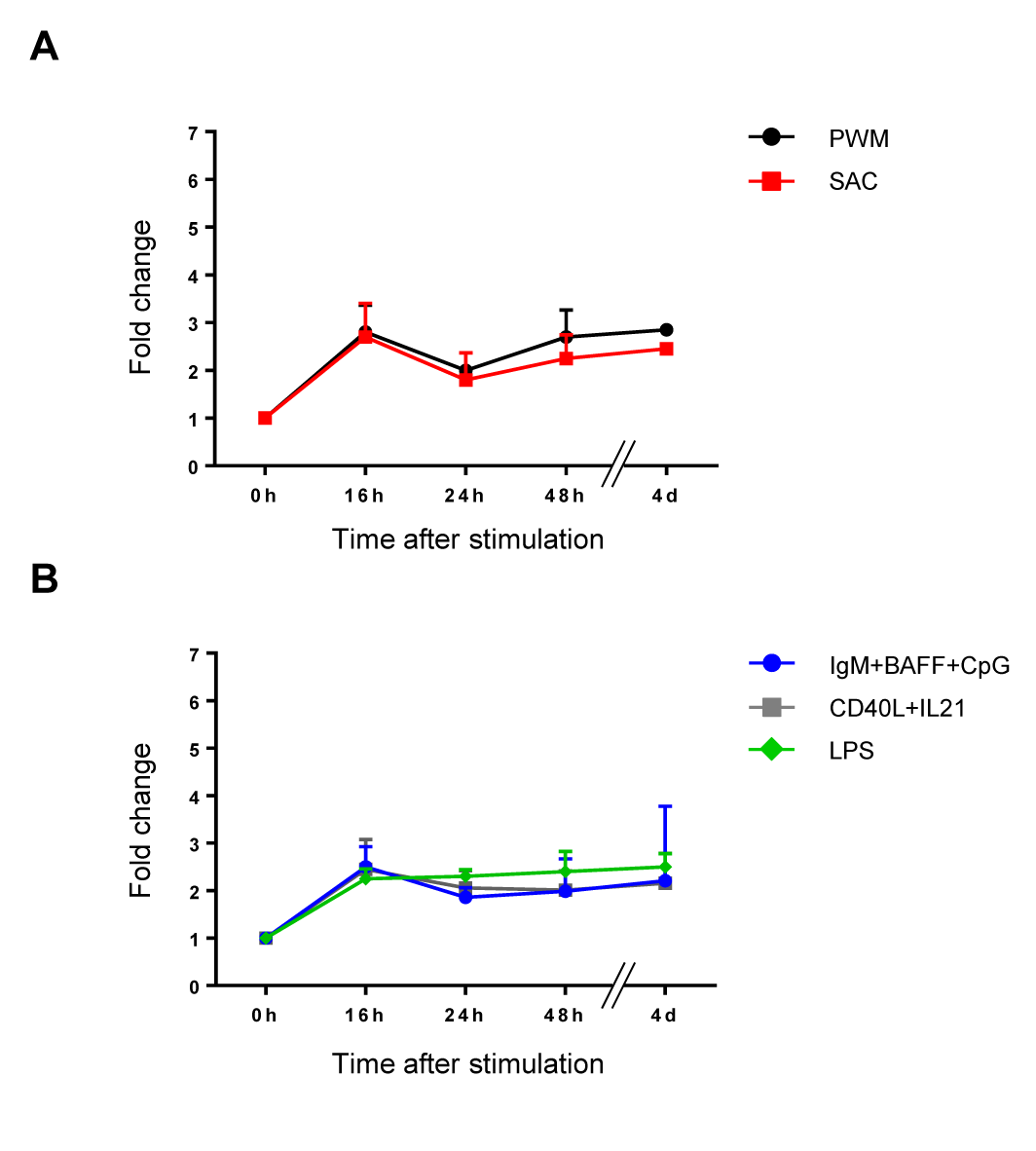

Supplement: Figure S2 — Time course of LRBA protein expression in total peripheral blood mononuclear cells (PBMCs) using different stimuli. Statistical analysis was performed comparing 0 h with 16, 24, 48 h, or 4 days after stimulation. All stimulation conditions and time points were performed in three samples independently. [file image_2.tif]

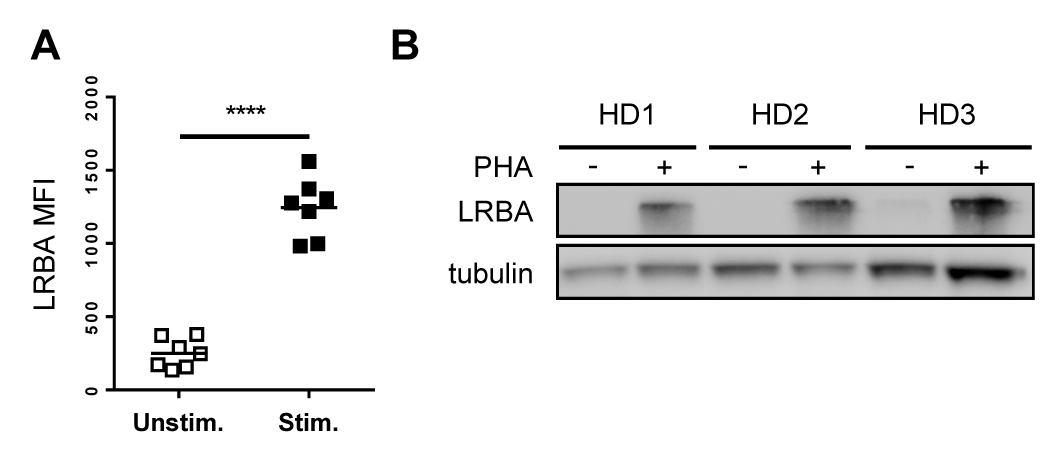

Supplement: Figure S3 — Peripheral blood mononuclear cells (PBMCs) need stimulation to promote LRBA expression. LRBA expression was detected by flow cytometry in fresh PBMCs from seven different healthy donors without stimulation (white squares) and upon stimulation with phytohaemagglutinin (PHA) (10 ng/µl) for 3 days (black squares) (A), or by western blotting in fresh PBMCs from three healthy donors before and after stimulation with PHA (B). [file image_3.tif]

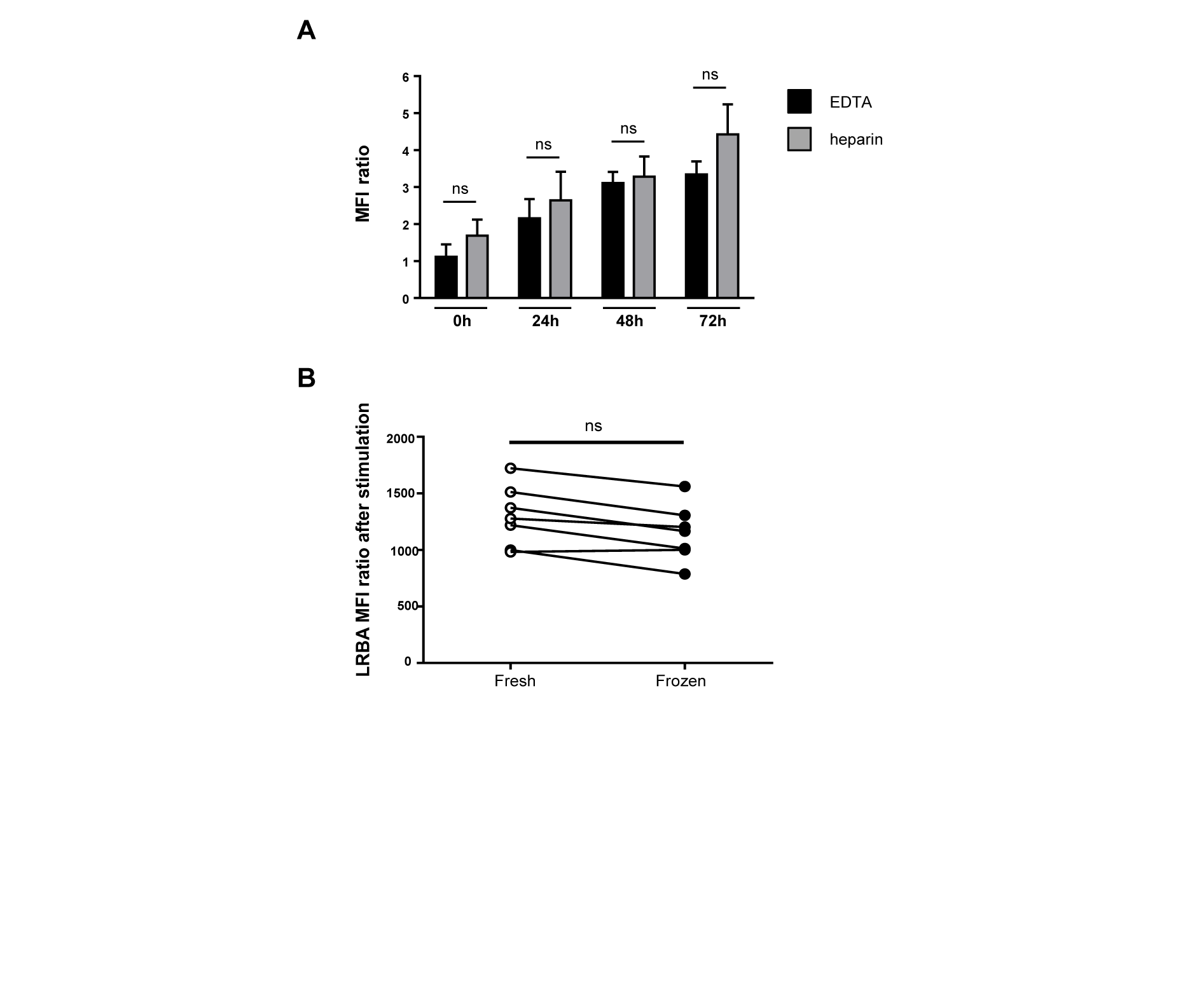

Supplement: Figure S4 — LRBA expression is not affected by (A) the type of anticoagulant use for blood withdrawal, by the time of withdrawal, or (B) by frozen storage. [file image_4.tif]

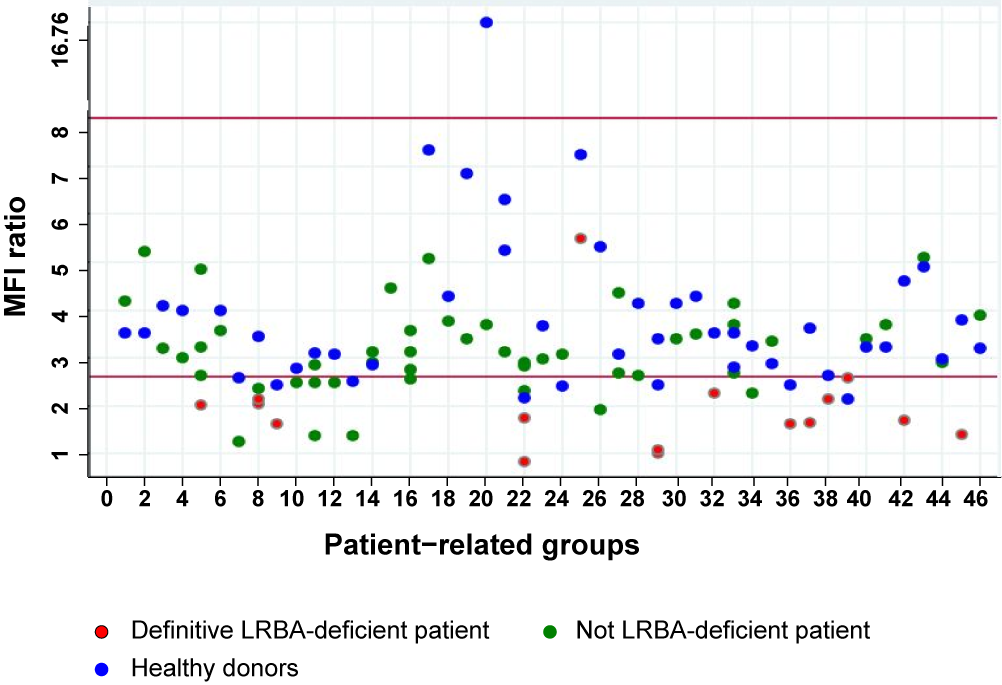

Supplement: Figure S5 — Scatter plots representing the LRBA-MFI ratio distribution of the different 16 definitive LRBA patients (red dots), and 41 non-LRBA patients (green dots) according to their respective healthy donors (blue dots). [file image_5.tif]
