# Supplementary material for: Rapid Flow Cytometry-Based Test for the Diagnosis of Lipopolysaccharide Responsive Beige-Like Anchor (LRBA) Deficiency
Source: Front Immunol. 2018 Apr 23;9:720. doi: 10.3389/fimmu.2018.00720 (PMC5925005; doi:10.3389/fimmu.2018.00720)
Supplement: Table S1 — Classification of patients with a suspicion of LRBA deficiency. [file table_1.PDF]

Supplementary Table 1. Classification of patients with a suspicion of LRBA deficiency

Group	Criteria
**Possible**	Patients who met the following criteria are considered as suspected of LRBA deficiency:<br><br>I. Unknown genetic cause of their disease and any of the following diagnosis: IIa. ALPS-phenotype (Autoimmune lymphoproliferative syndrome of undetermined genetic cause), IIb. CVID (Common Variable Immunodeficiency), IIc. Child-onset hypogammaglobulinemia, IId: early-onset Inflammatory Bowel Disease, i.e. age of onset <10 years, and IIe: Autoimmune cytopenias of unknown etiology. III. Signed patient consent. IV. Availability of samples.
**Probable**	Possible LRBA-deficient patients with absence or reduced LRBA protein expression tested by flow cytometry or Western blot, or as suggested here, with a LRBA-MFI ratio below 2.6
**Definitive**	Probable LRBA-deficient patients with biallelic mutations in *LRBA*
**Not LRBA-deficient**	Patients with a suspicion of LRBA deficiency but with wild type sequence of *LRBA*
