# Supplementary material for: Rapid Flow Cytometry-Based Test for the Diagnosis of Lipopolysaccharide Responsive Beige-Like Anchor (LRBA) Deficiency
Source: Front Immunol. 2018 Apr 23;9:720. doi: 10.3389/fimmu.2018.00720 (PMC5925005; doi:10.3389/fimmu.2018.00720)
Supplement: Table S2 — LRBA mutations identified in our patient’s cohort. [file table_2.PDF]

Supplementary Table 2. LRBA mutations identified in our patient´s cohort

Patient internal ID	*LRBA* mutation (cDNA sequence)	Inheritance
LRBA_1	c.2445_2447del(C)3ins(C)2	Homozygous
LRBA_2	c.2445_2447del(C)3ins(C)2	Homozygous
LRBA_3	c.8139_8142insCATG	Homozygous
LRBA_4	c.7937 A>C	Homozygous
LRBA_5	c.6191C>G	Heterozygous
	c.8164T>C	Heterozygous
LRBA_6	c.675G>A	Homozygous
LRBA_7	c.7162delA	Homozygous
LRBA_8	c.1073G>A	Homozygous
LRBA_9	c.1420G>A	Heterozygous
	c.2834_2837delTCTT	Heterozygous
LRBA_10	c.2450-3 C>A	Homozygous
LRBA_11	c.1787C>T	Heterozygous
	c. 2170A>G	Heterozygous
LRBA_12	c.1787C>T	Heterozygous
	c. 2170A>G	Heterozygous
LRBA_13	c.2455A>G	Homozygous
LRBA_14	c.2762C>G	Homozygous
LRBA_15	c.5143C>T	Heterozygous
	c.7337C>A	Heterozygous
LRBA_16	c.1787C>T	Heterozygous
	c. 2170A>G	Heterozygous
